# Incidence and Risk of Coronavirus Disease 2019 Hospitalization Among Unvaccinated Children

**DOI:** 10.1111/irv.70022

**Published:** 2024-10-21

**Authors:** Ousseny Zerbo, Julius Timbol, John R. Hansen, Kristin Goddard, Evan Layefsky, Pat Ross, Bruce Fireman, Dao Nguyen, Tara L. Greenhow, Nicola P. Klein

**Affiliations:** ^1^ Vaccine Study Center Kaiser Permanente Northern California Oakland California USA; ^2^ Department of Pediatrics Kaiser Permanente Northern California Oakland California USA; ^3^ Pediatrics Infectious Diseases Kaiser Permanente Northern California Oakland California USA

**Keywords:** COVID‐19, hospitalization, pediatric infections, unvaccinated

## Abstract

**Objectives:**

The aim of this study is to determine the incidence and risk factors associated with COVID‐19 hospitalization among unvaccinated children.

**Methods:**

Children aged 0– < 18 years, members of Kaiser Permanente Northern California (KPNC), were followed from March 1, 2020, until the earliest occurrence of: chart‐confirmed COVID‐19 hospitalization, disenrollment from KPNC, age 18 years, receipt of COVID‐19 vaccine, death, or study end (December 31, 2022). We calculated the incidence rate of hospitalization by SARS‐CoV‐2 variant period and by age group. We determined risk factors for hospitalization using Poisson regression. We also conducted descriptive analyses of hospitalized cases.

**Results:**

Among 1,107,799 children, 423 were hospitalized for COVID‐19 during follow‐up. The incidence of hospitalization increased with each new SARS‐CoV‐2 variant and was highest among children aged < 6 months. Among the < 6‐month‐olds, the incidence rate per 100,000 person‐months was 7 during predelta, 13.3 during delta, and 22.4 during omicron. Black (RR = 2.05, 95% CI: 1.33–3.16) and Hispanic children (RR = 1.82, 95% CI: 1.34–2.46) and children with any comorbidities were at high risk of hospitalization (RR = 3.81, 95% CI: 2.94–4.95). Overall, 20.3% of hospitalized children were admitted to an intensive care unit (ICU), but ICU admission was 36.1% among 12– < 18‐year‐olds. The majority of ICU admits (91.8%) had no comorbidities.

**Conclusion:**

Children too young to be vaccinated had the highest incidence of COVID‐19 hospitalization, while adolescents had the highest proportion of ICU admissions. To prevent severe disease in children and adolescents, everyone eligible should be vaccinated.

**Abbreviations**
ADHD: Attention‐deficit hyperactivity disorder
ASD: Autism spectrum disorder
CDC: Centers for Disease Control and Prevention
CI: Confidence interval
COVID‐19: Corona virus disease 2019
KPNC: Kaiser Permanente Northern California
ICU: Intensive care unit
MIS‐C: Multisystem inflammatory syndrome in children
RR: Risk ratio
SARS‐CoV‐2: Severe acute respiratory distress syndrome coronavirus 2

## 1 Introduction

Since the start of the coronavirus disease 2019 (COVID‐19) pandemic in December 2019 through May 2023, approximately 15.6 million children in the United States have tested positive for COVID‐19 [1]. This represents over 17% of all COVID‐19 cases in the United States. These numbers underrepresent the true number of infected cases since many children may have mild disease and fail to undergo SARS‐CoV‐2 testing or tested positive using home tests and were not reported. Seroprevalence data reported that SARS‐CoV‐2 infection is higher than those reported for children [2, 3, 4].

Although most children often experience mild illness following SARS‐CoV‐2 infection, children can develop severe disease requiring hospitalization, admission into an intensive care unit (ICU), or die [5, 6, 7, 8, 9]. In the weeks following COVID‐19, some may develop multisystem inflammatory syndrome in children (MIS‐C).

Following the introduction of COVID‐19 vaccines in December 2020 for persons 16 years and older, the number of severe COVID‐19 cases (hospitalization, ICU admission and death) among all ages decreased significantly [10]. However, after the emergence of the SARS‐CoV‐2 B.1.1.529 (Omicron) variant in December 2021, hospitalizations among children aged <5 years, who were ineligible for vaccination at that time, increased more rapidly than did those in other age groups [11]. During the earlier period of the Omicron variant, COVID‐19 hospitalization rates in infants aged <6 months were higher than those of all other age groups except adults aged ≥65 years [12]. The expansion of vaccination recommendations to adolescents 12–15 years in May 2021, children 5–11 years in November 2021, and to children 6 months to 4 years in June 2022 led to a decrease in severe cases in these groups [13].

Although several studies have shown that COVID‐19 vaccines are effective in preventing infection and severe disease among children [14, 15, 16, 17, 18, 19], vaccine coverage among children has been suboptimal compared to adults [20]. According to a recent study, approximately half (50.1%) of children aged < 18 years in the United States who are eligible for vaccination received at least 1 dose of COVID‐19 vaccine, and only 44.2% completed their primary vaccination series. By age group, primary series completion was 13.2% in children <5 years, 43.9% in children 5–11 years, and 63.3% in adolescents 12–17 years [21].

Although vaccination and natural immunity have altered the epidemiology of severe COVID‐19, we continue to see the appearance of new variants capable of infecting and causing disease. The objective of this study was to determine the incidence of COVID‐19 hospitalization among unvaccinated children in the context of widespread vaccine availability, to determine risk factors associated with hospitalization, and to describe clinical characteristics of cases at admission and medical procedures during hospital stay.

## 2 Methods

### 2.1 Study Design, Setting, and Population

The study was a retrospective cohort study conducted at Kaiser Permanente Northern California (KPNC), an integrated healthcare system with a stable population of approximately 4.5 million members. Among the members, about 1 million are aged <18 years. KPNC's clinical databases include comprehensive information about diagnoses in the outpatient, emergency department and inpatient settings, prescriptions and laboratory tests that are updated daily, and sociodemographic information. This study included children ages <18 years, who were members of Kaiser Permanente Northern California between March 1, 2020 and December 31, 2022.

### 2.2 Outcome

The outcome was hospitalization with a SARS‐CoV‐2 infection among unvaccinated children confirmed by reverse transcription‐polymerase chain reaction (PCR) test. Children who tested positive and were not hospitalized, those who tested negative and those who never tested were the comparative group referred to here as the nonhospitalized KPNC pediatric population.

We conducted medical chart reviews using a standardized review form to confirm that the hospitalization was related to COVID‐19 and not an incidental positive test among patients hospitalized for other reasons and to capture clinical presentation at hospital admission, proportion admitted to an ICU, diagnoses, procedures, and treatment during hospital stay, median length of stay and the discharge disposition. Children whose medical record indicated that they were hospitalized due to COVID‐19 were considered severe cases.

### 2.3 Demographic Factors and Comorbidities

Demographic factors examined included sex, race/ethnicity, and type of insurance (subsidized or nonsubsidized) as a proxy for socio‐economic status. Comorbidities were classified by International Classification of Diseases, Tenth Revision codes (ICD 10) and included attention‐deficit hyperactivity disorder (ADHD; ICD 10 F90 – F98, autism spectrum disorder (ASD; ICD 10 F80 – F89, cerebral palsy (ICD 10G80 ‐ G83), diabetes, epilepsy, immunocompromised status (ICD 10 D80 – D89), intellectual disability (ICD 10 F70 – F79), and sleep apnea (ICD 10, G47.3).

#### 2.3.1 Statistical Analysis

We followed children from March 1, 2020 until the earliest occurrence of the following: chart‐confirmed hospitalization for COVID‐19, disenrollment from KPNC, reached age 18 years, received COVID‐19 vaccine, death, or end of study (December 31, 2022). We calculated the monthly incidence rate of hospitalization by dividing the number of hospitalized cases by the number of person‐months at risk. Incidence rates were calculated by SARS‐CoV‐2 variant circulating period and by age group (<6 months, 6–11 months, 1 ‐ < 5 years, 5 ‐ < 12 years, 12 ‐ < 18 years). Based on California's variant circulation data [22], we defined the predelta period as from March 1, 2020 to June 19, 2021, the delta period from June 20, 2021 to December 20, 2021, and the omicron period from December 21, 2021 to December 31, 2022.

We conducted descriptive analyses of sociodemographic characteristics and comorbidities of hospitalized children and those of the nonhospitalized KPNC pediatric population. We used Poisson regression to determine sociodemographic and comorbidities associated with risk of hospitalization.

The KPNC Institutional Review Board approved and determined this study to be exempt from human subject review under federal regulations. We conducted all analyses with SAS version 9.4 (SAS Institute, Inc, Cary, North Carolina).

## 3 Results

The study included 1,107,799 children who contributed 22,147,167 person‐months of follow up. During follow‐up, we identified 899 children who were hospitalized with a SARS‐CoV‐2 PCR positive test. After review of electronic medical charts, 423 (47%) were confirmed to be hospitalized due to COVID‐19 and 476 (53%) were incidental cases. Of the chart‐confirmed hospitalized cases, 103 (24.3%) were <6 months of age, 40 (9.5%) were 6–11 months, 139 (32.9%) were between 1 and <5 years, 80 (18.9%) were between 5 and <12 years, and 61 (14.4%) were between 12 and <18 years of age (Table 1). For all ages, the incidence of hospitalization increased with each new SARS‐CoV‐2 variant. Incidence was highest among children aged < 6 months, with an incidence rate per 100,000 person‐months of 7.0 during the pre‐delta period, 13.3 during the delta period and 22.4 during the omicron period.

**TABLE 1:** Monthly incidence rates of COVID‐19 hospitalization among unvaccinated children by age group and by circulating SARS‐CoV‐2 variant. Kaiser Permanente Northern California, March 1, 2020 through December 31, 2022.

		Incidence^a^ of hospitalization by SARS‐CoV‐2 circulating time period					
	All period: March 1, 2020 to Dec. 31, 2022	Pre‐delta period: March 1, 2020 to June 19, 2021		Delta period: June 20, 2020 to Dec. 20, 2021		Omicron period: Dec. 21, 2021 to Dec. 31, 2022	
	*N* = 423	*N* = 105		*N* = 85		*N* = 233	
Age category	*n* (%)	*n* (%)	Incidence rate	*n* (%)	Incidence rate	*n* (%)	Incidence rate
<6 months	103 (24.3)	24 (22.9)	7.0	18 (21.2)	13.3	61 (26.2)	22.4
6–11 months	40 (9.5)	4 (3.8)	1.2	6 (7.1)	5.1	30 (12.9)	12.7
1 ‐ < 5 years	139 (32.9)	21 (20.0)	0.8	19 (22.4)	1.8	99 (42.5)	5.2
5 ‐ < 12 years	80 (18.9)	29 (27.6)	0.6	24 (28.2)	1.3	27 (11.6)	1.4
12 ‐ < 18 years	61 (14.4)	27 (25.7)	0.6	18 (21.2)	2.7	16 (6.9)	1.6

^a^ Incidence per 100,000 person‐months.

Socio‐demographically, the proportions of males (56.5% vs. 51.1%), subsidized insurance (28.1% vs. 17.5%), Black (8.5% vs. 5.6%),s and Hispanics (38.3% vs. 24.0%) were higher among the hospitalized children compared with the nonhospitalized KPNC pediatric population. However, the proportion of white children (25.1% vs. 27.9%) was lower among the hospitalized children than the nonhospitalized KPNC pediatric population (Table 2). Among the comorbidities, the proportions of children with sleep apnea (10.2% vs. 2.6%), ASD (4.5% vs. 2.8%), cerebral palsy (3.8% vs. 0.2%), diabetes (3.8% Vs. 0.3%), epilepsy (6.9% vs. 0.5%), and immunocompromised status (4.5% vs. 0.1%) were higher among the hospitalized than the nonhospitalized KPNC pediatric population (Table 2). In covariate‐adjusted analyses from a Poisson regression model, Black children (risk ratio [RR] = 2.05, 95% confidence interval [CI]: 1.33–3.16), and Hispanic children (RR = 1.82, 95% CI: 1.34–2.46) were at significantly higher risk of hospitalization compared with White children (Table 3). Children with any comorbidities were at higher risk of hospitalization compared with those without a comorbidity (RR = 3.81, 95% CI: 2.94–4.95) (Table 3). Among the individual comorbidities examined, only ADHD and ASD were not significantly associated with increased risk of hospitalization.

**TABLE 2:** Characteristics of unvaccinated children hospitalized for COVID‐19 and nonhospitalized children age <18 years.^a^ Kaiser Permanente Northern California. March 1, 2020 to December 31, 2022.

	Unvaccinated COVID‐19 hospitalized children *N* = 423	Nonhospitalized children *N* = 1,107,376
	*n* (%)	*n* (%)
Sex		
Female	183 (43.3)	540,432 (48.8)
Male	239 (56.5)	566,275 (51.1)
Other	1 (0.2)	37 (0.0)
Unknown	0 (0.0)	632 (0.1)
Type of insurance		
Nonsubsidized	254 (60.0)	692,725 (62.6)
Subsidized	119 (28.1)	194,006 (17.5)
Unknown	50 (11.8)	220,645 (19.9)
Race/ethnicity		
Asian	64 (15.1)	198,558 (17.9)
Black	36 (8.5)	61,827 (5.6)
Hawaiian/Pacific Islander	3 (0.7)	9097 (0.8)
Hispanic	162 (38.3)	266,118 (24.0)
Multiracial	12 (2.8)	28,213 (2.5)
Native American/Alaskan	1 (0.2)	2786 (0.3)
White	106 (25.1)	309,351 (27.9)
Unknown	39 (9.2)	231,426 (20.9)
Comorbidities		
Attention‐deficit hyperactivity disorder (ADHD)	24 (5.7)	73,904 (6.7)
Sleep apnea	43 (10.2)	29,096 (2.6)
Autism spectrum disorders (ASD)	19 (4.5)	31,330 (2.8)
Cerebral Palsy	16 (3.8)	1831 (0.2)
Diabetes	16 (3.8)	3090 (0.3)
Epilepsy	29 (6.9)	5527 (0.5)
Intellectual disability	11 (2.6)	2075 (0.2)
Immunocompromised status	19 (4.5)	1374 (0.1)
None of the above comorbidities	312 (73.8)	982,245 (88.7)

^a^ Includes children who tested positive or negative and not hospitalized and those not tested for COVID.

**TABLE 3:** Risk Factors associated with COVID‐19 hospitalization among unvaccinated children ages <18 years. Kaiser Permanente Northern California. March 1, 2020 to December 31, 2022.

Risk factors	Adjusted relative risk^a^ (95% CI)
Asian	1.15 (0.79–1.67)
Black	2.05 (1.33–3.16)
Hawaiian/Pacific Islander	1.52 (0.48–4.84)
Hispanic	1.82 (1.34–2.46)
Multiracial	1.41 (0.70–2.83)
Native American/Alaskan	1.75 (0.24–12.62)
Unknown	0.42 (0.22–0.80)
White	Reference
Attention‐deficit hyperactivity disorder	0.48 (0.267–0.87)
Sleep apnea	3.62 (2.35–5.56)
Autism spectrum disorders (ASD)	1.19 (0.66–2.15)
Cerebral Palsy	4.57 (1.89–11.04)
Diabetes	13.99 (7.51–26.07)
Epilepsy	5.79 (2.93–11.44)
Immunocompromised status	16.86 (7.90–35.98)
Intellectual disability	1.71 (0.59–4.90)
Any of the above comorbidities	3.81 (2.94–4.95)

^a^ Each variable was adjusted for the others.

At hospital admission, 344 (81.3%) children had fever, 198 (46.8%) had poor feeding, 235 (55.6%) had a cough, and 174 (41.1%) had shortness of breath or respiratory distress (Table 4).

**TABLE 4:** Clinical presentation at hospital admission, diagnoses, procedures, and treatments during hospitalization. Kaiser Permanente Northern California March 1, 2020 to December 31, 2022.

	All ages *N* = 423	Age groups				
		<6 months *N* = 103	6–11 months *N* = 40	1–< 5 years *N* = 139	5–11 years *N* = 80	12–< 18 years *N* = 61
	*n* (%)	*n* (%)	*n* (%)	*n* (%)	*n* (%)	*n* (%)
Clinical presentation at admission						
Fever	344 (81.3)	85 (82.5)	32 (80.0)	119 (85.6)	68 (85.0)	40 (65.6)
Poor feeding/anorexia	198 (46.8)	53 (51.5)	22 (55.0)	71 (51.1)	35 (43.8)	17 (27.9)
Cough	235 (55.6)	56 (54.4)	28 (70.0)	82 (59.0)	35 (43.8)	34 (55.7)
Diarrhea	90 (21.3)	14 (13.6)	12 (30.0)	27 (19.4)	29 (36.3)	8 (13.1)
Vomiting	149 (35.2)	21 (20.4)	15 (37.5)	59 (42.4)	34 (42.5)	20 (32.8)
Respiratory distress	174 (41.1)	31 (30.1)	24 (60.0)	62 (44.6)	28 (35.0)	29 (47.5)
Abdominal pain	71 (16.8)	1 (1.0)	0 (0.0)	25 (18.0)	32 (40.0)	13 (21.3)
Congestion/runny nose	173 (40.9)	51 (49.5)	26 (65.0)	67 (48.2)	16 (20.0)	13 (21.3)
Headache	55 (13.0)	1 (1.0)	1 (2.5)	9 (6.5)	25 (31.3)	19 (31.1)
Diagnoses during hospitalization						
Sore throat	51 (12.1)	2 (1.9)	2 (5.0)	14 (10.1)	20 (25.0)	13 (21.3)
Myocarditis/pericarditis	9 (2.1)	0 (0.0)	0 (0.0)	2 (1.4)	3 (3.8)	4 (6.6)
Multisystem inflammatory syndrome in children	67 (15.8)	2 (1.9)	2 (5.0)	26 (18.7)	29 (36.3)	8 (13.1)
Procedure and treatment during hospital stay						
Acute respiratory distress syndrome	7 (1.7)	0 (0.0)	1 (2.5)	2 (1.4)	3 (3.8)	1 (1.6)
Mechanical ventilation	17 (4.0)	2 (1.9)	1 (2.5)	8 (5.8)	3 (3.8)	3 (4.9)
Oxygen support without ventilation	164 (38.8)	22 (21.4)	20 (50.0)	61 (43.9)	32 (40.0)	29 (47.5)
Extracorporeal membrane oxygenation	1 (0.2)	0 (0.0)	0 (0.0)	0 (0.0)	1 (1.3)	0 (0.0)
Remdesivir treatment	98 (23.2)	12 (11.7)	9 (22.5)	23 (16.5)	24 (30.0)	30 (49.2)
Hospital length of stay (days): Median (IQR)	2 (1,3)	1 (1,2)	1 (0,2)	1 (0,3)	3 (2,5)	3 (1,5)
Admitted to an intensive care unit	86 (20.3)	7 (6.8)	7 (17.5)	31 (22.3)	19 (23.8)	22 (36.1)
Any comorbid condition among the ICU admits	39 (9.2)	0 (0.0)	1 (2.5)	14 (10.1)	14 (17.5)	10 (16.4)
Discharged disposition						
Discharged to home	419 (99.1)	103 (100.0)	40 (100.0)	138 (99.3)	78 (97.5)	60 (98.4)
Died	4 (0.9)	0 (0.0)	0 (0.0)	1 (0.7)	2 (2.5)	1 (1.6)

Among the hospitalized children, nine (2.1%) children were diagnosed with myocarditis/pericarditis and the highest proportion of myocarditis/pericarditis (6.6%) was among children ages 12– < 18 years. Also, 67 (15.8%) children were diagnosed with MIS‐C, and the highest proportion of MIS‐C (36.3%) was among children ages 5–11 years.

During hospitalization, 164 (38.8%) children received oxygen support without ventilation. This proportion varied by age group (Table 4). Among these children, 75 (46%) were treated with remdesivir.

Of the hospitalized children, 86 (20.3%) were admitted to an ICU. The highest proportion of which was among children ages 12– < 18 years (35.1%), and the lowest was among children ages <6 months (6.8%; Table 4). Among the ICU admits, 16 (18.6%) required mechanical ventilation. The majority of ICU admits (91.8%) had no comorbidities; only a small proportion of (9.2%) of ICU admits had a comorbid condition. Finally, four (0.9%) children died due to COVID‐19 complications (Table 4).

## 4 Discussion

In this large, population‐based study, the incidence of confirmed COVID‐19 hospitalization among unvaccinated children ages 0– < 18 years varied by age group and by circulating SARS‐CoV‐2 variant. The incidence of hospitalization was highest among children aged < 6 months, followed by those aged 6–11 months. Within each age group, the incidence of hospitalization increased with the occurrence of each new SARS‐CoV‐2 variant. These data suggest that younger children who are not eligible for vaccination are at the highest risk of hospitalization. One effective way to protect these younger children is through maternal vaccination during pregnancy which provides passive immunity to infants. We and others have shown that COVID‐19 vaccination during pregnancy is highly effective at protecting infants against SARS‐CoV‐2 infection and severe disease including hospitalization during the first 6 months of life [23, 24, 25, 26].

In this study, we found that children aged 12–17 years had the lowest incidence of hospitalization. However, they had the highest proportion of admission to an ICU and of requiring oxygen support during hospitalization. These results suggest that while older children might be less likely to be hospitalized compared to younger children, when they do get hospitalized, they require a higher level of care.

Previous studies have reported that the Omicron variant was more transmissible and does not cause severe disease among adults [27, 28, 29]. However, in this study, we found that the incidence of hospitalization increased with the occurrence of each variant and the incidence was highest during the Omicron period. We do not know whether children were more susceptible to severe disease or whether the high volume of infection led to more severe disease among children.

Like previous COVID‐19 studies in the general population [30, 31, 32] and among children [33, 34, 35, 36, 37], we also found that Black and Hispanic children and those with a comorbid condition had a higher risk of hospitalization. These results suggest that like adults, children from minority race/ethnic groups are disproportionally affected by severe COVID‐19.

Our results show that children hospitalized due to COVID‐19 can develop life‐threatening disease. We found that 20% of hospitalized children were admitted to an ICU, 16% were diagnosed with MIS‐C, 4% required mechanical ventilation, 2% were diagnosed with myocarditis, and 0.9% died. Among the 5–11‐year‐olds, 36% were diagnosed with MIS‐C. Our 20% ICU admission among those hospitalized was lower than that reported by a recent study which found 41% ICU admission among COVID‐19 hospitalization in children [38]. This high proportion may be due to the inclusion of some hospitalizations resulting in ICU admission not due to COVID‐19 because the study did not assess the reason for hospitalization as we did through medical record reviews.

Although we found that children with medical comorbidities were at higher risk of hospitalization than those without, only 9% of children admitted to an intensive care unit had a comorbidity. Contrary to popular belief that only children with comorbidities are at risk of severe COVID‐19, our data suggest that any child can be at risk of severe disease requiring intensive care because the proportion of comorbidities among the children admitted to an ICU was small and most children admitted to an ICU had no comorbidities. Although the World Health Organization recommends a risk based COVID‐19 vaccination for children [39], our results suggest that all children can benefit from vaccination to reduce their risk of hospitalization.

One of the most effective ways to prevent severe COVID‐19 in the general population has been vaccination [40, 41], which have been available for children as young as 6 months since June 2022 and for older children since May 2021. However, vaccine uptake in the pediatric population has been suboptimal. Previous studies reported that COVID‐19 symptoms are mild among vaccinated people compared with unvaccinated people [42, 43]. Although the present study did not compare hospitalization among vaccinated and unvaccinated people, the results provide further evidence that it is important for parents to vaccinate their children to prevent severe COVID‐19.

The study was strengthened by using longitudinal medical record data, which allowed us to follow the children until they were hospitalized for COVID‐19, or vaccinated, at which point they were censored. Thus, the results apply to children who were not vaccinated. The availability of medical charts allowed us to manually review all potential COVID‐19 hospitalized cases and confirm that the hospitalization was due to COVID‐19 symptoms instead of testing positive during a hospitalization unrelated to COVID‐19. The study was also strengthened by its large size and diverse race and ethnicity.

This study has limitations worth noting. The estimation of the incidence rate of hospitalization censored children when they were vaccinated. Therefore, the study results do not apply to the vaccinated population. The goal of this study was not to compare rate and disease severity between vaccinated and unvaccinated children. The goal was to determine and characterize rates of disease among unvaccinated only. Further studies are needed to do such comparison. We also only counted the first hospitalization by child and did not assess multiple hospitalizations. Although the study included a large population, there were too few cases to fully adjust the analysis beyond age, a limited set of comorbidities, and race/ethnicity. Similarly, despite the incidence of hospitalization increasing with each new variant, we were also not able to determine whether the incidence of ICU admission or other clinical conditions increased over time. The study was not able to disentangle whether infants <6 months with positive SARS‐CoV‐2 test were hospitalized for observation or whether they were hospitalized because of severe disease. The proportion of children hospitalized who received remdesivir was small, and the study was not able to determine why the majority of children who were hospitalized did not receive remdesivir. Finally, for children less than 6 months of age, we did not adjust the incidence of hospitalization for maternal COVID‐19 vaccination status.

## 5 Conclusion

The incidence of hospitalization among unvaccinated children increased over time with the appearance of new SARS‐CoV‐2 variants. Incidence was highest among children <6 months of age who are not eligible for current recommended vaccines. The proportion of complications requiring ICU admission was higher among children eligible for vaccination. To prevent severe disease in children and adolescents, everyone eligible including pregnant persons should be vaccinated.

## Author Contributions

**Ousseny Zerbo:** conceptualization, investigation. **Julius Timbol:** formal analysis, data curation. **John R. Hansen:** formal analysis. **Kristin Goddard:** administrative support and chart reviews, validation. **Evan Layefsky:** administrative support and chart reviews, methodology. **Pat Ross:** administrative support and chart reviews, methodology. **Bruce Fireman:** conceptualization, investigation. **Dao Nguyen:** supervision, provided clinical input. **Tara L. Greenhow:** supervision. **Nicola P. Klein:** conceptualization, investigation.

## Disclosure

Dr. Klein has received research support from Pfizer for COVID vaccine clinical trials and research support from GlaxoSmithKline, Sanofi Pasteur, Merck, Pfizer, and Protein Science (now Sanofi Pasteur) for unrelated studies.

## Conflicts of Interest

The authors declare no conflicts of interest.

### Peer Review

The peer review history for this article is available at https://www.webofscience.com/api/gateway/wos/peer‐review/10.1111/irv.70022.

## Data Availability

The datasets generated during and/or analyzed during the current study are not publicly available due to potentially identifiable information (e.g., dates of diagnoses) and KPNC privacy regulations. They are available from the corresponding author upon reasonable request and contingent on appropriate human subjects approval and data use agreements.
